# Realizing the “40 by 2022” Commitment From the United Nations High-Level Meeting on the Fight to End Tuberculosis: What Will It Take to Meet Rapid Diagnostic Testing Needs?

**DOI:** 10.9745/GHSP-D-19-00244

**Published:** 2019-12-23

**Authors:** Amy S. Piatek, William A. Wells, Kaiser C. Shen, Charlotte E. Colvin

**Affiliations:** a United States Agency for International Development, Washington, DC, USA.

## Abstract

The potential gains from full adoption of World Health Organization (WHO)-recommended rapid diagnostics (WRDs) for tuberculosis (TB) are significant, but there is no current analysis of the additional investment needed to reach this goal. We sought to estimate the necessary investment in instruments, tests, and money, using Xpert MTB/RIF (Xpert), which detects *Mycobacterium tuberculosis* (MTB) and tests for resistance to rifampicin (RIF), as an example. An existing calculator for TB diagnostic needs was adapted to estimate the Xpert needs for a group of 24 countries with high TB burdens. This analysis assumed that countries will achieve the case-finding commitments agreed to at the recent United Nations High-Level Meeting on the Fight to End Tuberculosis, and that countries would adopt the WHO-recommended algorithm in which all people with signs and symptoms of TB receive an Xpert test. When compared to the current investments in these countries, this baseline model revealed that countries would require a 4-fold increase in the number of Xpert modules and a 6-fold increase in the number of Xpert test cartridges per year to meet their full testing needs. The incremental cost of the additional instruments for these countries would total approximately US$474 million, plus an incremental cost each year of cartridges of approximately $586 million, or a 5-fold increase over current investments. A sensitivity analysis revealed a variety of possible changes under alternative scenarios, but most of these changes either do not meet the global goals, are unrealistic, or would result in even greater investment needs. These findings suggest that a major investment is needed in WRD capacity to implement the recommended diagnostic algorithm for TB and reach the case-finding commitments by 2022.

## Abstract (precis)

Existing rapid diagnostics offer faster and more sensitive diagnosis of tuberculosis (TB) and simultaneous detection of multidrug-resistant TB. A 5-fold increase in investment in these tools is needed to meet the needs of the TB community and the United Nations’ ambitious 40 million by 2022 diagnosis and treatment target.

## INTRODUCTION

We are at a time of unprecedented attention and opportunity for tuberculosis (TB). At the United Nations High-Level Meeting on the Fight To End Tuberculosis on September 26, 2018,^1^ world leaders committed to bold targets and urgent action to end TB, including diagnosing and treating a cumulative 40 million people by 2022 (40 by 2022).^2^ This commitment cannot be met without equally bold and urgent responses to the greatest challenges facing national TB programs including the continued lack of access to quality and rapid TB diagnostics for people with signs and symptoms of TB. In 2017, 3.6 million people with TB went either undiagnosed or were detected and not reported, representing 36% of the estimated 10 million new cases.^3^

Rapid and accurate diagnosis is a critical requirement for an effective TB care and prevention effort^4^ because delayed diagnosis results in greater morbidity and mortality and increased disease transmission. For too long, TB programs had access only to smear microscopy, a century-old technology that has low sensitivity, detecting only about half of all TB cases (fewer in paucibacillary disease) and not detecting drug-resistant TB at all.^5^^,^^6^ Although we now have rapid TB diagnostic tests, the majority of people in high TB burden countries continue to be tested for TB with smear microscopy.^3^

Multiple documents from the global TB community have outlined the target of universal access to World Health Organization (WHO)-recommended rapid diagnostics (WRDs). Pillar 1 of the End TB Strategy^7^ states that early diagnosis of all persons of all ages with any form of TB is fundamental and that WRDs and drug susceptibility testing should be available to all who need it. WHO recommends that TB programs use WRDs that allow for the simultaneous detection of TB and drug-resistant TB as the initial diagnostic test instead of smear microscopy (e.g., in the compendium of WHO guidelines and standards^8^; see methods section for further details). Finally, the Global Laboratory Initiative’s Model TB Diagnostic Algorithms,^9^ originally published in early 2017 and recently revised, provides the preferred algorithm for universal patient access to rapid testing to detect *Mycobacterium tuberculosis* (MTB) and resistance to the anti-TB drug rifampicin (RIF). The algorithm currently indicates that the initial diagnostic test to use is the Xpert MTB/RIF (Xpert) assay, including for use with adults and children with signs and symptoms or chest X-ray (CXR) suggestive of TB, with persons being evaluated for extrapulmonary TB, and with persons being evaluated for TB who are HIV-positive.

Multiple documents from the global TB community have outlined the target of universal access to WHO-recommended rapid diagnostics.

Initial roll-out of Xpert began in the second quarter of 2010, and by the end of that year, 18 countries had 166 modules in place and cumulatively had run approximately 27,000 tests (Philippe Jacon, Cepheid, email communication, November 2018). By the end of 2017, 130 countries had procured 'more than 42,000 modules and 34.4 million tests.^3^ However, in countries where data were available and reported, only 20.6%^10^ of new and relapse cases were tested using WRDs in 2017.

Effective TB case finding first requires a variety of approaches to identify all people with signs and symptoms of TB.^11^^–^^13^ Although this initial identification of presumptive TB is critically important to reaching the 40 by 2022 targets, the focus of the current study is to investigate whether or not TB diagnostic networks in high-burden countries have the capacity to provide rapid and accurate testing for an initial TB diagnosis for these clients and to determine the actual requirements to test all people with signs and symptoms of TB with a WRD. This effort requires a realistic calculation of what volumes of testing and numbers of testing instruments a country needs. We present such an analysis using Xpert as an example because it is the most widely implemented WRD currently (although the lessons should apply equally to other WRDs).^14^

## METHODS

We adapted the WHO TB diagnostic capacity calculator^15^ (WHO calculator) to generate estimates of the need for rapid TB diagnostic testing instruments and assays using the Xpert instrument and Xpert MTB/RIF cartridges as an example. We estimated the volume of Xpert cartridges needed to test persons presenting with signs and symptoms of TB and the number of Xpert modules that are needed to provide full coverage for TB diagnosis in a high-burden country under realistic operating conditions. The baseline model calculated the cartridges and modules needed to identify 90% of the total estimated TB cases (all forms)^3^ because this reflects the level of case finding needed to reach the 40 by 2022 targets, as innovative case finding approaches are implemented at scale. The analysis was conducted for the 24 countries currently receiving direct United States Agency for International Development (USAID) funding for TB^16^; these countries represent 74% of the world’s TB burden.

The WHO calculator was originally developed using a stakeholder consensus process and consists of a simple but logical Microsoft Excel format.^15^ Populations who require testing are calculated from WHO epidemiological information; these figures are further multiplied based on the projected usage of Xpert. Adapting the WHO calculator methodology, which dates from a period of more conservative use of Xpert in diagnostic algorithms, the calculation was performed to determine the rapid TB diagnostic testing needs for 3 populations of TB patients: (1) HIV-negative adults; (2) HIV-positive adults; and (3) children (0–14 years old). WHO data were used to estimate the number of HIV-negative adults and the number of children with TB,^10^ in both cases multiplied by 90% to reflect the 40 by 2022 targets, as noted above. The Joint United Nations Programme on HIV/AIDS (UNAIDS) 2017 data^17^ were used to estimate the total number of people living with HIV (PLHIV) for each country, which was then multiplied by 81% (the number of PLHIV enrolled on antiretroviral therapy [ART] if 90% of all PLHIV know their status and 90% of all who know their status are enrolled on ART, as expected under the UNAIDS 90-90-90 treatment targets^18^). We assume that, at each of 2 visits per year, 20% of those enrolled on ART will be eligible for Xpert testing based on a symptom screen (see later justification for the 20% value).

Realistic operating capacity per module was defined as an instrument that runs 168 days per year (70% of a full working year, accounting for facility closure due to holidays or worker actions, absence of staff, or compromised power supply) and 3 tests per day (accounting for delays in specimen transport, stock-out of cartridges, staff workload, and limited operating hours).

The model, and in particular the choice of populations assumed to require Xpert, conforms to the guidance and standards found in the WHO compendium.^8^ This includes Standard 6, which states that “all patients with signs and symptoms of pulmonary TB who are capable of producing sputum should have as their initial diagnostic test at least 1 sputum specimen submitted for Xpert MTB/RIF Ultra assay,” and Standard 5, which confirms that “TB programmes should transition to replacing microscopy as the initial diagnostic test with WHO-recommended rapid diagnostics that allow for the simultaneous detection of TB and drug-resistant TB.” Due to this assumption that all persons with signs and symptoms of TB would receive an Xpert test, the percentage of individuals that would get an Xpert test solely to test for resistance to rifampicin and not primarily for case detection was set at 0. Of note, the calculations are not expected to differ depending on use of Xpert MTB/RIF or Xpert Ultra because the algorithm would remain the same and the cost of MTB/RIF and Ultra cartridges is the same for high-burden countries. The WHO calculator’s additional calculation for relapse patients was also omitted because our overall calculation was based on testing 90% of all estimated new and relapse patients, but the WHO variable “ret_nrel” (previously treated patients, excluding relapse cases) was retained. For the purposes of this analysis, the WHO calculator does not estimate any use of the Xpert instrument for other indications such as HIV viral load testing, early infant diagnosis of HIV, and/or hepatitis C testing.

Systematic reviews were conducted in both Google and PubMed for terms including (“TB” OR “tuberculosis”) AND (“NNT” OR “NNS” OR “number needed to treat” OR “number needed to screen” OR “TB testing” OR “TB screening”); these results were also refined by adding search terms such as “HIV” and “X-ray.” The limited relevant data resulting from this search are described in the results. Based on these findings, in the baseline model, testing 10 persons with signs and symptoms of TB with Xpert yields on average 1 diagnosed TB patient (see Results for justification). The equivalent number used for children was 4 tested with Xpert to yield 1 TB patient, based on the defaults used in the original WHO calculator.

These baseline model parameters (see Table 1) were then varied in a sensitivity analysis to cover a range of possible country-specific values and scenarios. This exercise was limited to a deterministic sensitivity analysis because the paucity of available evidence would not support the use of uncertainty distributions or of multivariate models—the latter were judged more likely to obscure rather than illuminate the critical findings.

**TABLE 1.** Baseline Model Parameters Needed to Calculate Xpert Cartridges and Modules to Identify 90% of Total Estimated TB Cases

**Parameter**	**Value**
Estimated TB burden coverage	90%
Type of WRD	Xpert MTB/RIF
Who receives a WRD?	All with TB symptoms
Number of adults with symptoms needed to test with WRD to diagnose one adult with TB	10
Number of children with symptoms needed to test with WRD to diagnose one child with TB	4
Number of days per year that WRD (module) is operational	168
Number of test cycles per day per module	3
Estimated percentage of PLHIV enrolled on ART	81%
TB screening visits per year for clients on ART	2
Percentage of clients on ART with symptoms that require WRD	20%

Abbreviations: ART, antiretroviral therapy; PLHIV, people living with HIV; TB, tuberculosis; WRD, World Health Organization-recommended rapid diagnostic.

Given the limited geographical access to Xpert testing in almost all high-burden countries, we created a separate calculation (unrelated to the WHO calculator described above) to illustrate the potential demand for Xpert based on population size, regardless of estimated burden of TB and TB/HIV coinfection and operational capacity. We converted the standard for access to smear microscopy described under the Global Plan to Stop TB^19^ (1 microscope per 100,000 population) to the corresponding number of Xpert sites that would be needed to provide the same level of geographic coverage. This exercise was limited to an estimation of the minimum number of Xpert sites per country and did not consider the number of Xpert modules needed per site because that would have required using variables for the number of smears per day and the reasons for those smears, and reliable data to inform such a calculation were not available.

Finally, after generating the number of modules needed in the baseline model and various scenarios, we compared these outputs to the actual numbers of modules in countries and the numbers of test cartridges procured in 2017. Because WHO does not collect these data variables as part of their annual reporting and no other standardized database exists with this information, we used alternative data sources. For the number of modules currently in countries, we surveyed national TB program staff, USAID technical representatives, and other technical partners, and compared these figures to those from other relevant country reports. For the number of test cartridges available annually in countries, we used 2017 procurement data provided by Philippe Jacon, Cepheid, manufacturer of Xpert (email communication, November 2018). The Cepheid procurement data include test cartridges procured in the public sector for 145 high-burden and developing countries^20^ for MTB/RIF and MTB/RIF Ultra TB assays.

For the cost analysis, the current number of modules was subtracted from the total modules needed according to the baseline model to obtain the incremental number of modules required. This number was then divided by 4 and multiplied by the concessional cost of a 4-module machine with laptop (US$17,500 ex works, which does not include shipping or any potential customs costs; available to all of the high-TB burden countries included in this study). In line with the overall conservative approach to this analysis, variable service and maintenance costs were not included, and we used the global concessional price per test cartridge of US$9.98 ex works.

## RESULTS

### Determining Baseline Inputs

Below are detailed findings from the literature searches used to derive 3 of the model inputs.

#### Number of Adults With Symptoms Needed to Test With Xpert to Diagnose 1 Adult TB Patient

For the number of adults needed to test to diagnose 1 adult TB patient (number needed to test, or NNT), a 10:1 ratio has appeared in the guidance for many years.^21^ This ratio was originally based on expert consensus with the anticipation that countries would revise based on country-specific data because it varies with epidemiology and the intensity of case finding efforts. Based on this prior use and the evidence from South Africa (see below), a ratio of 10:1 was also used in the current model. Note, however, that WHO^15^^,^^22^ used a ratio of 10 tests to diagnose 1 bacteriologically positive patient (not 1 TB patient). This is a more complicated solution because the percentage of bacteriological positivity is expected to change over time with the increasing use of more sensitive diagnostics such as Xpert and Xpert Ultra, and we were not able to determine an evidence base for WHO’s rationale.

A clear country example would assist in justifying this important ratio. However, evidence from many countries was found to be focused on number needed to screen (NNS, the number of individuals that need to be asked about TB symptoms to diagnose 1 TB patient) instead of the NNT figure required as an input to the baseline model. In addition, most countries are still implementing a mixture of smear microscopy and Xpert, so it becomes difficult to untangle the number needed to test with Xpert to find 1 TB patient. South Africa is more promising in this regard because it uses Xpert as the primary diagnostic test for TB.^23^ Initial findings showed that the test positivity rate in South Africa jumped from the 8% seen with microscopy to 16%–18% in the first year of Xpert implementation, but this gradually declined to 12% in the fourth year,^24^ and in recent years has settled on 10.2% over multiple years of measurement.^25^

These South Africa numbers represent the ratio for detecting all TB, whether in PLHIV or HIV-negative individuals. Using the U.S. President's Emergency Plan for AIDS Relief (PEPFAR)^26^ and WHO^10^ data from 2017 and the first half of 2018, these data can be disaggregated into an estimated 7.6% of the Xpert testing volume being used to screen PLHIV (yielding approximately 29% of the total TB cases with NNT of 2.6 due to the non-aggressive symptom screening, which also explains why only 29% of case finding was in PLHIV despite a coinfection rate of 60%), compared to an NNT of 13.7 for the remaining 61% of diagnosed TB patients. Overall, this programmatic experience continues to support using a ratio of at least 10 adults tested with Xpert to 1 diagnosed TB patient, with the caveat that NNT varies substantially depending on prevalence (lower prevalence means higher NNT), symptom screening algorithm (a more inclusive symptom screen means higher NNT), and clinical practice.

Programmatic experience supports using a ratio of at least 10 adults tested with Xpert to 1 diagnosed TB patient.

#### Percentage of PLHIV on ART With Signs and Symptoms of TB Who Require Xpert

The percentage of PLHIV on ART who should be tested with Xpert varies considerably between what is ideal (and seen in study settings) versus what is typically done programmatically.

WHO recommends that PLHIV should be routinely screened for active TB at every health facility visit using a 4-symptom screen (current cough–any duration, fever, night sweats, and weight loss).^27^ The presence of any 1 of these 4 symptoms is considered a positive screen, and the absence of all 4 symptoms is considered a negative screen. Thus, the current definition of optimal practice is to be broad and inclusive in the symptom screen. In countries, however, the exact symptom screen used varies, and also the patients' definition of a cough and the providers’ index of suspicion vary. Thus, it is not possible to get a single, consistent number for this percentage, not just because of varying epidemiology but also because of this between-country variation.

A meta-analysis^28^ includes summary statistics of 11% with cough of 2 weeks or more, 20% of PLHIV with current cough, and 47% with any 1 of current cough, fever, night sweats, or weight loss (the latter being the WHO-recommended definition of symptomatic TB among PLHIV).

In some more recent individual studies, the percentage of PLHIV or client on ART judged to have a positive symptom screen that warranted TB testing varied by country setting:

- from 5% in Ghana, in a setting with an unusually restrictive algorithm requiring cough plus 1 other symptom^29^
- to 10.5% in India, though 30% actually had at least 1 TB symptom^30^
- to 20.9% in Ghana, though only 12.6% before an intervention to increase provider awareness^31^
- to 22.9% in Rwanda, a high-screen positive percentage despite a relatively high median CD4+ of 385^32^
- to 39% in Ethiopia, despite 89% being on ART^33^
- to 53% in Kenya, screened at enrollment, including 25% with current cough^34^

Meanwhile, global PEPFAR programmatic data showed a value of only 2.8% in PEPFAR countries based on PEPFAR data from Panorama for the first half of 2018 (Sevim Ahmedov, MD, USAID, email communication, November 2018), presumably based on incomplete implementation and a low index of suspicion.

Based on this evidence, and because the model is aiming for optimal practice in terms of coverage and implementation, the baseline model includes a value for this variable of 20% (representing a value that is the median of the 3 values from the meta-analysis and is very close to the median from the 6 more recent studies). Because 20% is probably still lower than optimal, and actual implementation is closer to 3%, the sensitivity analysis also includes scenarios that cover the range of 2.5% to 30% for this variable.

#### Percentage of People With TB Signs and Symptoms and an Abnormal CXR Requiring Xpert

Based on WHO guidance,^35^ “CXR and further clinical assessment can be used to triage who should be tested with the Xpert MTB/RIF assay to reduce the number of individuals tested and the associated costs, as well as to improve the pretest probability for TB and, thus, the predictive value of the Xpert MTB/RIF assay.” Therefore, at least in theory, CXR could be used in this model to reduce the number of Xpert modules and tests needed.

We estimate that about 50% of people with signs and symptoms of TB will have any abnormality on CXR sufficiently suggestive of TB to merit further evaluation. This assumption would halve the number of Xpert cartridges and modules needed, but only if it was possible to develop CXR capacity at the subdistrict level to facilitate patient access—a massive task, and one that would increase resource needs in other ways.

The best data to support the 50% estimate would be a survey of actual CXRs from the country among people with TB symptoms. However, prevalence surveys do not typically report their data in terms of “number of people with symptoms who had an abnormal CXR.” The primary input to the estimate of 50%, which was originally based on expert opinion, is from a single study that found that 45% of people with symptoms in Kenya had an abnormal CXR as read by a primary physician.^36^

### Baseline Model Outputs

Table 2 presents the logic of the main calculations, and Table 3 presents the results of the baseline model. The total current number of modules in the 24 countries was 26,873 (average per country of 1,120; range 80–4,780), whereas the total modules needed in the baseline scenario was 135,198 (average per country of 5,633; range 138–49,986), suggesting the need for a 4-fold increase in the number of Xpert modules across these countries, with the percentage increase needed per country ranging from 13% (Tajikistan) to 946% (India).

**TABLE 2.** Calculation Logic for Baseline Model

**Value to calculate**	**Components used in calculation**	**Formulae used**
Total annual number of Xpert MTB/RIF tests = Number of tests for HIV+ adults, children, and HIV− adults	Number of tests for HIV+ adults	Estimated number of PLHIV^a^ · 81%^b^ · 2^c^ · 20%^d^
	Number of tests for children	Estimated number of children with TB^e^ · 90%^f^ · 4^g^
	Number of tests for HIV− adults	Number of TB patients^e,^^h^ · 90%^f^ · Percentage of TB patients who are adults^e^ · Percentage of adults who are HIV−^a^ · 10^i^
Target number of Xpert modules needed		Total annual number of Xpert MTB/RIF tests/68^j^ · 3^k^

Abbreviations: HIV+, HIV positive; HIV−, HIV negative; MTB, *Mycobacterium tuberculosis*; TB, tuberculosis; WRD, World Health Organization-recommended rapid diagnostic.

a Source: Joint United Nations Programme on HIV/AIDS.

b Target percentage of persons living with HIV who are enrolled on ART (90%· 90%).

c Number of TB screening visits per year for clients on ART.

d Percentage of clients on ART with symptoms that require testing with a WRD.

e Source: World Health Organization.

f Target for TB-burden coverage.

g Number of symptomatic children needed to test with WRD to diagnose 1 child with TB.

h All forms, including all incident, relapse, and previously treated.

i Number of symptomatic adults needed to test with WRD to diagnose 1 adult with TB.

j Number of days per year that WRD module is operational.

k Number of test cycles per day per module.

**TABLE 3.** Xpert Modules and Test Cartridges Needed Under Baseline Model for 24 High-Burden Countries, by Country

**Country**	**Current Number of Modules**	**Total Modules Needed**	**Change Needed from Current (%)**	**No. Test Cartridges (2017)**	**No. Test Cartridges Needed, Annually**	**Change Needed from Current (%)**
Afghanistan	180	1,146	537%	17,500	577,600	3201%
Bangladesh	860	6,229	624%	341,900	3,139,400	818%
Cambodia	300	898	199%	134,050	452,700	238%
Democratic Republic of the Congo	614	4,368	611%	32,350	2,201,700	6706%
Ethiopia	1268	3,111	145%	203,950	1,567,700	669%
India	4780	49,986	946%	2,543,150	25,192,700	891%
Indonesia	2356	14,545	517%	507,450	7,330,800	1345%
Kenya	838	2,914	248%	450,450	1,468,900	226%
Kyrgyzstan	80	167	108%	16,200	84,000	419%
Malawi	428	873	104%	62,150	440,100	608%
Mozambique	368	3,061	732%	150,250	1,542,900	927%
Myanmar	367	3,120	750%	41,300	1,572,400	3707%
Nigeria	1576	8,182	419%	349,850	4,123,500	1079%
Pakistan	2808	9,087	224%	435,050	4,579,900	953%
Philippines	1436	10,029	598%	301,200	5,054,500	1578%
South Africa	4204	7,027	67%	2,198,000	3,541,500	61%
Tajikistan	122	138	13%	133,150	69,600	−48%
Tanzania	852	2,866	236%	395,250	1,444,400	265%
Uganda	994	1,762	77%	300,850	888,000	195%
Ukraine	292	725	148%	80,000	365,200	357%
Uzbekistan	208	453	118%	76,100	228,100	200%
Vietnam	690	2,230	223%	219,500	1,124,000	412%
Zambia	720	1,190	65%	166,850	599,700	259%
Zimbabwe	532	1,092	105%	247,900	550,300	122%
TOTAL	26,873	135,198	403%	9,404,400	68,139,600	625%

Many modules are currently operating at a lower capacity than that assumed in the baseline model; therefore, the cartridge gap (the gap between the number of test cartridges procured in 2017 and the total number needed) was even greater than the module gap (the gap between the current number of modules and the number needed). A more than 6-fold increase in test cartridges would be needed to get from the current volume per year in the 24 countries of 9,404,400 (average per country of 391,850 cartridges per year; range: 16,200–2,543,150) to the baseline model total need of 68,139,600 (average per country of 2,839,150 cartridges per year; range: 69,600–25,192,700), with the percentage increase per country ranging from −48% (Tajikistan) to 6706% (Democratic Republic of the Congo).

Under the baseline model, a more than 6-fold increase in the volume of test cartridges would be needed.

### Sensitivity and Scenario Analysis

The results of a sensitivity and scenario analysis are presented in Table 4. The full data for this analysis are listed by country in a Supplement. The changes that would, on average, decrease the number of modules needed include the scenarios that use 2017 actual TB case notifications instead of 90% of the estimated burden (leading to a total reduction of 32%), test a lower percentage of PLHIV on ART (leading to a total reduction of 6%–10%), assume a higher module operating capacity (48% reduction), or assume availability and use of CXR to triage persons with symptoms of TB before the WRD (50% reduction). The scenarios resulting in an increase in the number of modules needed above the baseline model include countries with lower TB prevalence, more ambitious TB case finding (with up to 100% or more increase in module needs from baseline), and more ambitious TB screening of PLHIV (6% increase in modules needed). The likelihood of these various scenarios is explored further in the Discussion.

**TABLE 4.** Sensitivity Analysis Relative to the Baseline Model

**Scenario Name**	**Parameter to Change**	**Default Value**	**Effect of Variation on Model Output**
1. Current notifications	Estimated TB burden	90%	If estimated TB burden is reduced to show only the capacity needed for current TB notifications, this will reduce the number of modules needed by a total of 32% (range: 8%–55%).
2. Reduced TB prevalence	Number of adults with symptoms needed to test with rapid diagnostic (Xpert) to diagnose 1 adult TB patient (NNT)	10	Number of needed modules changes almost proportionately (e.g., increasing to 12 tests will increase output by up to 19%). As prevalence decreases, the value will increase.
3. More screening of PLHIV	Percentage of PLHIV on ART with signs and symptoms of TB that require Xpert test	20%	Increasing the percentage to 30% will increase number of modules needed by a total of 6% (range: 0%–38%).
4. Less screening of PLHIV			Decreasing to 10% will decrease number of modules needed by a total of 6% (range: 0%–38%).
5. Current screening of PLHIV			Decreasing to 2.5% will decrease number of modules needed by an average of 10% (range: 0%–67%).
6. Increased operation of module	Operational capacity	168 days/year 3 cycles/day	Increasing working days to 240 and test throughput to 4 cycles/day will reduce number of modules needed by 48% in all countries.
7. CXR triage	Number needed to test	10	Including CXR as a triage tool before the WRD is estimated to reduce the number needed to test to 5 for HIV-negative adults and 2 for children, and to reduce the baseline number of PLHIV on ART requiring Xpert testing by 50%; in total, this would therefore reduce the number of modules and cartridges needed by 50%. See text for justification.
8. Ambitious case finding	Number needed to test and operational capacity	10 for NNT; 3 cycles/day	To detect all people with TB, more ambitious case finding is needed. This is likely to result in both more down-time for modules (due to greater decentralization and/or using mobile screening, thus cycles/day is reduced to 2) and a lower positivity rate from testing more people with symptoms of TB (thus NNT is increased to 20). This combination of changes increases modules and cartridges needed by 177% and 84% (range: 80%–194% and 20%–96%).
9. WRD sites	Access standard for smear microscopy	None	Converts access standard for smear microscopy (1 microscope/100,000 population) to WRD sites needed to achieve same geographical coverage. This produces a large number of sites needed, though these values are more than 4 times lower than the baseline modules needed, since each site will require multiple modules to achieve sufficient throughput.

Abbreviations: CXR, chest X-ray; NNT, Number needed to test; PLHIV, people living with HIV; TB, tuberculosis; WRD, World Health Organization-recommended rapid diagnostic.

The numbers of WRD sites to mimic the 1 microscope per 100,000 population requirement used for smear microscopy access are a total of 78% (range 30%–92%) smaller than the baseline values for number of modules, indicating that many of the sites would likely have more than a single module to achieve the baseline scenario (Supplement). Indeed, the number of these sites required under the 1 microscope per 100,000 population calculation is only 13% more, on average, than the current number of modules in these countries (range −87% in South Africa to 180% in India).

### Cost Analysis

The cost implications of the baseline model are presented in Table 5. The baseline model would require a total incremental investment across the 24 countries of US$473,920,210 (average per country of $19,746,675; range $70,417–$197,774,132) in Xpert instruments, and $586,177,296 per year in cartridges (average per country of $24,424,054; range −$634,229–$226,042,059), for a total incremental investment of $1,060,097,504 (average per country of $44,170,729; range −$563,812 to $423,816,641). This represents a 5-fold increase over current investment in these countries because the current investment (all machines procured to date, plus the number of cartridges procured in 2017) is approximately $211,425,287 (average per country of $8,809,386; range $511,676–$46,293,137).

**TABLE 5.** Cost Implications of Baseline Model (All Values in US$)

**Country**	**A: Cost of Additional Xpert Modules (Based on the Price of a 4-Module Instrument)**	**B: Cost of 1 Year’s Supply of Additional Cartridges, According to Total Calculated Need**	**C: Total Incremental Cost of Baseline Model Over Current Situation (New Modules Plus 1 Year of Cartridges) (A+B)**	**D: Total Current Cost (Existing Modules Plus Number of Cartridges Procured in 2017)**	**E: Total % Increase in Investment Needed (C/D)**
Afghanistan	$4,226,389	$5,589,798	$9,816,187	$962,150	1020%
Bangladesh	$23,489,236	$27,919,050	$51,408,286	$7,174,662	717%
Cambodia	$2,617,188	$3,180,127	$5,797,315	$2,650,319	219%
Democratic Republic of the Congo	$16,425,729	$21,650,113	$38,075,842	$3,009,103	1265%
Ethiopia	$8,061,007	$13,610,225	$21,671,232	$7,582,921	286%
India	$197,774,132	$226,042,509	$423,816,641	$46,293,137	916%
Indonesia	$53,327,917	$68,097,033	$121,424,950	$15,371,851	790%
Kenya	$9,084,618	$10,164,131	$19,248,749	$8,161,741	236%
Kyrgyzstan	$379,167	$676,644	$1,055,811	$511,676	206%
Malawi	$1,947,813	$3,771,941	$5,719,754	$2,492,757	229%
Mozambique	$11,783,229	$13,898,647	$25,681,876	$3,109,495	826%
Myanmar	$12,043,681	$15,280,378	$27,324,059	$2,017,799	1354%
Nigeria	$28,899,271	$37,661,027	$66,560,298	$10,386,503	641%
Pakistan	$27,471,076	$41,365,603	$68,836,679	$16,626,799	414%
Philippines	$37,593,368	$47,437,934	$85,031,302	$9,288,476	915%
South Africa	$12,349,688	$13,408,130	$25,757,818	$40,328,540	64%
Tajikistan	$70,417	$(634,229)	$(563,812)	$1,862,587	-30%
Tanzania	$8,810,694	$10,470,517	$19,281,211	$7,672,095	251%
Uganda	$3,359,583	$5,859,757	$9,219,340	$7,351,233	125%
Ukraine	$1,892,639	$2,846,296	$4,738,935	$2,075,900	228%
Uzbekistan	$1,070,035	$1,516,960	$2,586,995	$1,669,478	155%
Viet Nam	$6,738,194	$9,026,910	$15,765,104	$5,209,360	303%
Zambia	$2,055,729	$4,319,843	$6,375,572	$4,815,163	132%
Zimbabwe	$2,449,410	$3,017,952	$5,467,362	$4,801,542	114%
TOTAL	$473,920,208	$586,177,296	$1,060,097,504	$211,425,287	
AVERAGE	$19,746,675	$24,424,054	$44,170,729	$8,809,387	474%

The baseline model would require a 5-fold increase in total investment.

## DISCUSSION

The TB diagnostics network is the foundation for all other interventions needed to end the global TB epidemic. Without an accessible, quality network of rapid TB diagnostics, countries and the global TB community will never reach the 40 by 2022 goal.

Without an accessible, quality network of rapid TB diagnostics, countries and the global TB community will never reach the 40 by 2022 goal.

We did an estimation exercise to determine if countries have adequate rapid TB testing capacity to be able to detect all people with TB, in line with the 40 by 2022 goal. This analysis showed that there is a considerable gap between the existing rapid testing capacity and the capacity that is actually needed. Compared to the current situation, the baseline model required a 4-fold expansion in Xpert module capacity across these 24 countries with high TB burdens, and a 6-fold increase in the number of test cartridges. The estimated total cost for this scale-up is approximately $474 million for additional modules, plus an incremental cost each year of cartridges of approximately $586 million. Proportionately, even greater needs are possible in countries with more active case finding, more aggressive screening of PLHIV, usage of Xpert machines for other diseases,^37^ or lower TB prevalence (because countries would need more tests to find the same number of TB cases).

These large volumes in the baseline model are consistent with early predictions of substantial potential market sizes for new TB diagnostics, with the prediction that 59% adoption of a smear-replacement test by 2020 would result in an estimated annual volume of 49 million tests.^38^ By comparison, the actual procurement reported in 2017 by Cepheid across all high-TB burden countries was less than a quarter of that, at 11.2 million tests procured (Philippe Jacon, Cepheid, email communication, November 2018).

For various reasons, a total required volume lower than that identified from the baseline model is possible (Table 4), but not likely. First, the ratio of people with TB symptoms to diagnosed TB patients may be less than 10:1, though some of the best data for this come from South Africa and point to, if anything, a higher number. Second, using the national TB program as the data source for Xpert module numbers may miss modules that have been procured directly by the private sector with their own private funding. However, the numbers of such machines in TB high-burden countries are minimal (and zero in many high-burden countries).^39^ Third, the use of CXR as a triage tool before the WRD could potentially approximately halve the WRD needs, though cost and major patient access issues around CXR have resulted in limited usage of such an algorithm.

Indeed, it may be optimistic to estimate an approximate 50% reduction in Xpert testing volume based on adding a CXR triage step. In the TB prevalence survey in Vietnam, 3.7% of the general population had a CXR abnormality,^40^ in a population where TB prevalence was 260 per 100,000 (0.26%), so the ratio of CXR abnormality to confirmed TB was greater than 10 to 1 and thus the NNT in a CXR triage algorithm remained high. Practicality of this algorithm is also a concern. In terms of resource needs, as a first approximation we could assume the same number of CXR machines being needed as the number of Xpert sites under the “per 100,000 population” calculation (see Supplement). Because a CXR takes only a few minutes, throughput reasons would likely not justify a greater number of CXR machines than Xpert machines. However, for patient accessibility, such an assumption is very much on the low end because it is far easier to transport sputum to an Xpert than patients to a CXR. Thus, this estimated need for CXR under this scenario should be considered a low-end estimate.

Fourth and finally, operational capacity of WRD machines might improve (less down-time and more cycles per day), though this is not what we see from current experiences in high-burden countries where there is increasing evidence for “Xpert for all” algorithms being incompletely implemented due to resource constraints. For example, Ethiopia has been aggressive in adopting an “Xpert for all” algorithm, which has almost tripled the use of Xpert in 3 years, but the peak utilization is still only 93%^41^ of the “realistic operating capacity” defined above (see Methods) or 54% of the original WHO implementation recommendations (3–4 tests/module/day · 250 days per year).^42^ This less than optimal operating capacity is not because of a lack of need; the percentage of TB cases tested for rifampicin resistance in these project areas has increased but is still only 28%.^41^ Indonesia represents another example, where substantial support for an “Xpert for all” algorithm in focus districts has resulted in more than double the use of Xpert, but peaking at only 38% of full operational capacity in focus districts (compared to 16% in non-supported provinces, using the WHO implementation recommendations).^43^ Thus, even with substantial support and an expansive algorithm, the instrument’s maximum potential operating capacity seems out of reach and the capacity presented in the baseline model presents a more realistic scenario.

Two other ways to reach lower numbers for the resource needs would be to assume either current case finding numbers (instead of the targeted 90% of total incident cases) and the current, inadequate TB screening percentages for PLHIV. Although in Table 4 we present the results of using such sensitivity analyses, our baseline model was aimed explicitly at estimating the future needs of the TB community to reach goals that have already been set. Both of these 2 sensitivity analyses go against that theme by settling for the status quo. Thus, although these 2 analyses are included in Table 4 for the sake of completeness, we do not see them as a challenge to the baseline numbers.

It is important to note that the number of Xpert tests needed for PLHIV screening does not decrease greatly with the widespread adoption of ART. Even PLHIV on ART will still have significant levels of TB symptoms from non-TB causes, just like the general population, and it is these non-TB causes that are behind the vast majority of the symptoms that prompt an Xpert test. The subsequent TB yield from those Xpert tests will decrease for people on ART, but the need for the tests in the first place remains, which is the relevant issue for this exercise.

When Xpert was introduced in 2010, it was intended to be a point-of-care test to replace smear microscopy. By doing this, countries would keep access intact but significantly improve sensitivity and be able to diagnose rifampicin resistance with the initial test. In reality, roll-out has been steady but slow.^44^^,^^45^ In 2013, WHO policy^46^ recommended that Xpert be used as the initial diagnostic test in adults and children suspected of having multidrug-resistant TB or HIV-associated TB; the use of Xpert as the initial test in all adults and children with symptoms of TB was a conditional recommendation and not taken up by most countries. Although the WHO Compendium^8^ states that Xpert is to be used as the initial diagnostic test in everyone with symptoms of TB (see Methods), the original restricted policy led to an uneven and slow uptake of Xpert as the primary diagnostic for all people with symptoms of TB. By the end of 2017, national algorithms and policies in only 32 of the 48 countries included in WHO’s lists of high TB, TB/HIV, and multidrug-resistant TB burden countries had been revised to include this recommendation for use of Xpert for all individuals with TB symptoms,^3^ and the extent of implementation in countries with these policies varies.

Why are WRDs including Xpert not being used universally as the primary diagnostic tool for TB? Beyond the explanation of insufficient financial resources, there are several plausible reasons.^47^ Originally, Xpert was intended to be a “near” point-of-care diagnostic placed within subdistrict facilities similar to the level of smear microscopy services; however, limited resources and operational challenges like unstable power supply forced Xpert to become more centralized and ultimately inaccessible without specimen transport mechanisms in place.^48^ Some of the operational barriers^48^^,^^49^ are slowly being addressed, including the use of alternative power sources like solar and diagnostic data management solutions like GXAlert/ASPECT that provides visibility to the program on all instruments (thus enabling a response to instrument problems or commodity issues). There is also an abundance of training material for all levels of the health system that can be used to build staffing capacity and there have been creative approaches to address staff shortages. But, despite all these interventions, the ability to move Xpert to the level of the microscopy center is still in doubt in many high-burden countries. Issues such as power, infrastructure (i.e., air conditioning), capacity of staff to troubleshoot, lack of maintenance and service, and module failures remain major operational challenges that will have to be faced in any ongoing expansion, with a focus on supporting the systems required for a true point-of-care functionality.^50^^,^^51^ In addition, since concessional pricing for the Xpert instrument and test cartridges is limited to the public sector, the test is mostly unavailable to persons who seek care from private providers and facilities.^39^

### Limitations

Our analysis has a number of limitations. There is limited information to inform the setting of values for certain key variables, including NNT. True numbers will, in any case, vary substantially between countries depending on epidemiology, the intensity of case finding, and other factors. Attempts to incorporate such considerations via stratification of the model would result in a less transparent and more questionable model based on suppositions rather than evidence and was therefore not undertaken. However, we believe that the baseline model remains a reasonable estimate that errs on the side of conservatism. For the estimation of current cartridge procurement volumes, the estimated values may not reflect true consumption if the procurement order for a country is not based on the previous year’s consumption due to leftover stock from the previous year or an increase or decrease in funding available for procurement. In addition, the resource needs estimate does not include a number of additional and substantial areas of investment that would be needed including the cost of maintenance contracts, shipping and customs payments, connectivity installment and maintenance, sputum transportation, infrastructure requirements, and training and paying salaries for additional staff. Finally, the required investment amount may differ if different WRDs, with a different price, are used instead of Xpert,^14^ or if Xpert machine or cartridge procurement is by private providers without access to concessional pricing.

## CONCLUSION

Even as countries continue to work out WRD expansion and operational issues, the issue of the total capacity needed (as addressed here) also remains, including the gap in the resources needed to reach that capacity. Rapid test availability is very far from the only issue and need that is confronting TB programs as they aim for the 40 by 2022 targets; there are a multitude of additional activity, financial, and system constraints that must also be addressed. However, it is clear from this analysis that countries do not have enough rapid TB test instruments or cartridges to meet their needs. Without increasing both instrument and cartridge numbers, countries will struggle to find all people with TB and to implement quality TB diagnostics at scale. Ambitious goals such as the 40 by 2022 require bold interventions. This includes urgently expanding access to and capacity of country TB diagnostic testing networks.

## Supplementary Material

19-00244-Wells-Supplement-ed.docx

## Notes

**Peer Reviewed**

**First Published Online:** December 5, 2019

**Funding:** No specific funding for this work.
