# Supplementary material for: Realizing the “40 by 2022” Commitment From the United Nations High-Level Meeting on the Fight to End Tuberculosis: What Will It Take to Meet Rapid Diagnostic Testing Needs?
Source: Glob Health Sci Pract. 2019 Dec 23;7(4):551–63. doi: 10.9745/GHSP-D-19-00244 (PMC6927833; doi:10.9745/GHSP-D-19-00244)
Supplement: 19-00244-Wells-Supplement-ed.docx [file 19-00244-Wells-Supplement-ed.docx]

**Supplement Table: Full Data on the Xpert Module and Cartridge Needs under the sensitivity analysis scenarios**

The following table provides the data outputs from the scenarios outlined in Table 4. The number of each scenario described below is listed in the first column of Table 4. For each scenario, all parameters were held constant (as outlined in Table 1) except for the single parameter described in the second column of Table 4, which was altered as described in the fourth column of Table 4.

| Country | Baseline Module Needs | Baseline Tests Needs | Scenario 1 Modules | **Scenario 1 Tests** | Scenario 2 Modules | **Scenario 2 Tests** | Scenario 3 Modules | **Scenario 3 Tests** | Scenario 4 Modules | **Scenario 4 Tests** | Scenario 5 Modules | **Scenario 5 Tests** | Scenario 6 Modules | **Scenario 6 Tests** | Scenario 7 Modules | **Scenario 7 Tests** | Scenario 8 Modules | **Scenario 8 Tests** | Scenario 9 # Sites |
| --- | --- | --- | --- | --- | --- | --- | --- | --- | --- | --- | --- | --- | --- | --- | --- | --- | --- | --- | --- |
| Afghanistan | 1,146 | 577,600 | 382 | 192,600 | 1,365 | 687,800 | 1,146 | 577,600 | 1,146 | 577,600 | 1,146 | 577,600 | 602 | 577,600 | 573 | 288,800 | 3,359 | 1,128,700 | 355 |
| Bangladesh | 6,229 | 3,139,400 | 2,699 | 1,360,200 | 7,423 | 3,741,300 | 6,233 | 3,141,500 | 6,225 | 3,137,300 | 6,222 | 3,135,700 | 3,270 | 3,139,400 | 3,114 | 1,569,700 | 18,300 | 6,148,700 | 1647 |
| Cambodia | 898 | 452,700 | 256 | 129,100 | 1,060 | 534,100 | 920 | 463,600 | 877 | 441,900 | 861 | 433,700 | 472 | 452,700 | 449 | 226,400 | 2,559 | 859,800 | 160 |
| Democratic Republic of the Congo | 4,368 | 2,201,700 | 1,897 | 956,200 | 5,145 | 2,593,000 | 4,494 | 2,264,800 | 4,243 | 2,138,500 | 4,149 | 2,091,100 | 2,293 | 2,201,700 | 2,184 | 1,100,900 | 12,376 | 4,158,200 | 813 |
| Ethiopia | 3,111 | 1,567,700 | 1,171 | 590,300 | 3,626 | 1,827,300 | 3,307 | 1,666,600 | 2,914 | 1,468,900 | 2,767 | 1,394,800 | 1,633 | 1,567,700 | 1,555 | 783,900 | 8,529 | 2,865,800 | 1050 |
| India | 49,986 | 25,192,700 | 18,696 | 9,422,700 | 59,392 | 29,933,800 | 50,661 | 25,532,900 | 49,311 | 24,852,500 | 48,804 | 24,597,400 | 26,242 | 25,192,700 | 24,993 | 12,596,300 | 145,531 | 48,898,500 | 13392 |
| Indonesia | 14,545 | 7,330,800 | 4,272 | 2,152,900 | 17,303 | 8,720,800 | 14,748 | 7,432,900 | 14,343 | 7,228,800 | 14,191 | 7,152,200 | 7,636 | 7,330,800 | 7,273 | 3,665,400 | 42,503 | 14,281,000 | 2640 |
| Kenya | 2,914 | 1,468,900 | 1,588 | 800,200 | 3,274 | 1,650,300 | 3,397 | 1,711,900 | 2,432 | 1,225,900 | 2,071 | 1,043,700 | 1,530 | 1,468,900 | 1,457 | 734,400 | 7,072 | 2,376,100 | 497 |
| Kyrgyzstan | 167 | 84,000 | 72 | 36,100 | 198 | 99,600 | 169 | 85,200 | 164 | 82,700 | 162 | 81,800 | 88 | 84,000 | 83 | 41,900 | 482 | 162,000 | 60 |
| Malawi | 873 | 440,100 | 711 | 358,500 | 917 | 462,000 | 1,195 | 602,100 | 552 | 278,100 | 311 | 156,600 | 458 | 440,100 | 437 | 220,100 | 1,635 | 549,400 | 186 |
| Mozambique | 3,061 | 1,542,900 | 1,702 | 857,700 | 3,371 | 1,698,800 | 3,736 | 1,883,100 | 2,386 | 1,202,700 | 1,880 | 947,600 | 1,607 | 1,542,900 | 1,531 | 771,400 | 6,912 | 2,322,500 | 297 |
| Myanmar | 3,120 | 1,572,400 | 901 | 454,200 | 3,683 | 1,856,000 | 3,190 | 1,608,000 | 3,049 | 1,536,700 | 2,996 | 1,510,000 | 1,638 | 1,572,400 | 1,560 | 786,100 | 8,901 | 2,990,700 | 534 |
| Nigeria | 8,182 | 4,123,500 | 3,217 | 1,621,200 | 9,338 | 4,706,300 | 9,178 | 4,625,700 | 7,185 | 3,621,300 | 6,438 | 3,244,700 | 4,295 | 4,123,500 | 4,091 | 2,061,800 | 20,945 | 7,037,400 | 1909 |
| Pakistan | 9,087 | 4,579,900 | 2,657 | 1,339,300 | 10,804 | 5,445,100 | 9,135 | 4,604,200 | 9,039 | 4,555,600 | 9,003 | 4,537,400 | 4,771 | 4,579,900 | 4,543 | 2,289,900 | 26,506 | 8,906,000 | 1970 |
| Philippines | 10,029 | 5,054,500 | 2,205 | 1,111,100 | 11,924 | 6,009,800 | 10,051 | 5,065,500 | 10,007 | 5,043,500 | 9,991 | 5,035,300 | 5,265 | 5,054,500 | 5,014 | 2,527,200 | 29,260 | 9,831,300 | 1049 |
| South Africa | 7,027 | 3,541,500 | 5,693 | 2,869,500 | 7,452 | 3,755,800 | 9,341 | 4,707,900 | 4,713 | 2,375,100 | 2,977 | 1,500,300 | 3,689 | 3,541,500 | 3,513 | 1,770,700 | 13,730 | 4,613,300 | 567 |
| Tajikistan | 138 | 69,600 | 59 | 29,800 | 163 | 81,900 | 143 | 72,000 | 133 | 67,100 | 130 | 65,300 | 73 | 69,600 | 69 | 34,800 | 391 | 131,300 | 89 |
| Tanzania | 2,866 | 1,444,400 | 1,340 | 675,200 | 3,234 | 1,629,900 | 3,348 | 1,687,400 | 2,384 | 1,201,400 | 2,022 | 1,019,200 | 1,505 | 1,444,400 | 1,433 | 722,300 | 7,058 | 2,371,600 | 573 |
| Uganda | 1,762 | 888,000 | 1,149 | 579,000 | 1,935 | 975,000 | 2,180 | 1,098,600 | 1,344 | 677,400 | 1,031 | 519,500 | 925 | 888,000 | 881 | 444,000 | 3,937 | 1,322,800 | 429 |
| Ukraine | 725 | 365,200 | 419 | 211,100 | 834 | 420,300 | 802 | 404,000 | 647 | 326,300 | 589 | 297,100 | 380 | 365,200 | 362 | 182,500 | 1,907 | 640,700 | 442 |
| Uzbekistan | 453 | 228,100 | 161 | 80,900 | 533 | 268,500 | 469 | 236,600 | 436 | 219,700 | 423 | 213,400 | 238 | 228,100 | 226 | 114,000 | 1,280 | 430,000 | 319 |
| Viet Nam | 2,230 | 1,124,000 | 1,135 | 571,800 | 2,623 | 1,321,800 | 2,311 | 1,164,500 | 2,150 | 1,083,500 | 2,089 | 1,053,100 | 1,171 | 1,124,000 | 1,115 | 562,000 | 6,289 | 2,113,000 | 955 |
| Zambia | 1,190 | 599,700 | 837 | 421,800 | 1,276 | 642,900 | 1,543 | 777,900 | 836 | 421,500 | 571 | 287,900 | 625 | 599,700 | 595 | 299,800 | 2,429 | 816,000 | 171 |
| Zimbabwe | 1,092 | 550,300 | 927 | 467,200 | 1,135 | 572,100 | 1,510 | 760,900 | 674 | 339,700 | 361 | 181,800 | 573 | 550,300 | 546 | 275,200 | 1,962 | 659,200 | 165 |
